# Advances in Bovine Immunology – New Tools and New Insights to Tackle Old Foes

**DOI:** 10.3389/fimmu.2015.00071

**Published:** 2015-02-17

**Authors:** Kieran G. Meade

**Affiliations:** ^1^Animal and Bioscience Research Department, Animal and Grassland Research and Innovation Centre, Teagasc, Trim, Ireland

**Keywords:** mastits, *M. bovis*, MAP, one health, immunobiotics, bovine immunology, pregnancy, miRNAs, tuberculosis

The United Nations predicts that the current world population of 7.2 billion is projected to reach 9.6 billion by 2050, which can only mean one thing for global agriculture, and that is increased pressure on production. One step to increase food supply is the abolition of the milk quota restrictions in the EU in 2015. It is inevitable with expansion and intensification of production that risks associated with disease will be exacerbated. This will have critical consequences for the sustainability of farming systems, for the safety of the food chain, and for human health. In this context, it is appropriate that we redouble every effort to understand the immune response, particularly to recalcitrant infectious diseases in cattle.

The publication of the bovine genome in 2009 and the advent of new high-throughput technologies have facilitated a massive expansion in our knowledge of the immune response in cattle. Genomic selection means we can now identify animals with superior genetics at birth and use them as parents of the next generation, thereby rapidly increasing the rate of genetic gain. While these methods are currently in use to breed cattle with superior genetics for production traits, they are not yet available to improve disease resistance. Mycobacterial and mammary gland (Mastitis) infections represent two diseases that severely impact global cattle production, with an annual estimated cost of $3 billion for TB globally (1) and multiples of this value for mastitis, especially when the costs associated with subclinical infection are included (2, 3). Both these diseases, as well as complementary analyses on the regulation of bovine immunity, are addressed by cutting edge papers in this edition of *Frontiers*.

*Mycobacterium tuberculosis* causes TB in human beings and over one-third of the world’s population are infected with this disease. Similarly, in cattle, related mycobacterial species cause potentially zoonotic infections, which are endemic in many parts of the world, despite stringent global surveillance and control programs. The advent of Next-Generation Sequencing (NGS) technologies holds significant promise to overcome limitations in current generation test sensitivity and specificity and as discussed by McLoughlin et al., transcript biomarker signatures have been identified, which discriminate between TB patients with active and latent disease. Indeed, McLoughlin et al. used multi-dimensional scaling to unambiguously classify peripheral blood leukocyte transcriptomic profiles from *Mycobacterium bovis* infected and healthy cattle, thereby uncovering potential biomarkers for *M. bovis* infection (4). This technology is a powerful tool for unraveling the complexities of host immune response and provides new layers of information, which deepens our understanding of host–pathogen interactions that underlie Mycobacterial disease pathogenesis. The macrophage is recognized as the key effector cell driving anti-mycobacterial immunity but which can be hijacked by mycobacteria, thereby contributing to suboptimal bacterial clearance and disease chronicity. Using the macrophage as a model, RNA-seq was performed after challenge with *Mycobacterium avium* subspecies *paratuberculosis* (MAP), which has uncovered novel genes that have not previously been associated with the host response to MAP infection (5). One of the key challenges with NGS technologies is the bioinformatic analysis to extract meaningful and biologically relevant findings from the wealth of data generated. Sophisticated system-biology tools have been employed by Killick et al. to generate biological interaction networks that usefully identify key hub and bottleneck genes that are central to the immune response and thereby potential targets for immunomodulation – either naturally by pathogens in their eternal quest for survival, or therapeutically with the development of new intervention strategies (6). Furthermore, a comparative analysis of the macrophage response to various strains of mycobacteria has been reviewed; *M. bovis* induced a distinct transcriptional profile in monocyte-derived macrophages compared to the more similar profiles of both *M. bovis* BCG and MAP (7). The authors describe how differential expression of type-I interferon genes were specific to the virulent *M. bovis* strain supporting a role for these genes in the establishment of active tuberculosis in cattle.

The identification of the genes and pathways involved in orchestration of the immune response is not merely of fundamental importance but differentiating between the immune responses to closely related bacterial species can have very real implications for the success of current generation diagnostics. The study by Kennedy et al., examines the effects of annual mandatory testing for *M. bovis* infection on the ELISA performance routinely used for the diagnosis of MAP (8). In one herd, prior to testing for bovine tuberculosis (BTB), 7.9% of cattle serum samples and 5.8% of milk samples were positive for MAP antibodies. Shortly after the BTB test, the MAP ELISA positive rate increased to 39% in both sample types, clearly showing BTB test interference in MAP ELISA performance. Exploiting differences in host immunity induced in response to these closely related strains of mycobacteria could yield significant dividends in terms of increased specificity of diagnostics.

An optimal and effective host immune response must overcome pathogen-mediated efforts to subvert it while minimizing tissue damage and, therefore, the regulation of the immune response is critical. Furthermore, shedding light on the differential dialog between the host and pathogenic or comensal bacterial strains is a very exciting area of research. It is of interest, therefore, that Villena et al. review how immunobiotics can dampen TLR-mediated inflammation in an intestinal cell line, and postulate how immunoregulatory feeds could be developed in the future (9). Inflammation is a feature of most diseases, and detailed knowledge on the pathways regulating it is critical toward developing effective immunoregulatory approaches in cattle. MiRNAs have also been shown to be powerful regulators of immunity and as reviewed by Lawless et al., 793 miRNAs have been identified to date in the bovine genome (10). Their rapid induction in response to challenge and the tissue-specific expression pattern of some has led to speculation on their potential use as diagnostics. Work by the same group has identified miRNA signatures of infection in mammary epithelial cells in response to a common mastitis-causing pathogen. The review highlights relevant studies on how miRNAs regulate the production of IFN-y and TNF, key cytokines in the immune response to TB.

In a comprehensive review, Thompson-Crispi et al. integrates multiple studies on the genetic regulation of the bovine immune response, particularly in relation to mastitis (11). Interestingly, the review discusses earlier work by the same group in which their High Immune Response (HIR) technology was used to identify Immunity^+^ sires, daughters of which showed a 44% reduction in mastitis as well as reduced susceptibility to other diseases. The potential consequences of selection for a specific immune phenotype are discussed, and the review signposts how integration of complementary genetic, genomic, and epigenetic data – supplemented with accurate disease and health phenotype information – will enhance our ability to breed cattle with superior disease resistance in the future.

Early fetal mortality is a major contributory factor to poor reproductive outcomes and increased costs, especially in high producing dairy cows. In that regard, the review by Fair is a relevant one. Although immunological analyses in the cow during pregnancy are growing, attempts to evaluate the interaction between the cow and the developing fetus are few. While comparative immunology can shed some light, Fair argues that basic understanding in the bovine are required to more comprehensively understand the complex regulation of local and systemic immunity in the pregnant cow and thereby yield novel solutions to fertility problems (12). Understanding the immune shifts that occur during pregnancy is also critical to understanding the windows of susceptibility that may exist through which susceptibility to infectious diseases could be increased.

Multi-factorial challenges, for example achieving and sustaining excellent animal health, require multifaceted solutions that can only be achieved through intensive integration of knowledge and expertise from a diverse spectrum of research efforts (13). As the physicist Richard J. Feynman once wrote “In order to make progress, one must leave the door to the unknown ajar.” There is a lot yet to learn in relation to bovine immunity and, therefore, this e-book is a timely integration of the most current research and scientific thinking on these critical issues and will contribute to the direction of future research in these areas. When dealing with infectious diseases, the old truism is perfectly apt – *ipsa scientia potestas est*. New knowledge also prepares us for the unforeseen challenges of the future.

## Conflict of Interest Statement

The author declares that the research was conducted in the absence of any commercial or financial relationships that could be construed as a potential conflict of interest.
